# Fluid homeostatic action of dapagliflozin in patients with chronic kidney disease: the DAPA-BODY Trial

**DOI:** 10.3389/fmed.2023.1287066

**Published:** 2023-12-14

**Authors:** Kentaro Oka, Takahiro Masuda, Ken Ohara, Marina Miura, Masato Morinari, Kyohei Misawa, Yasuharu Miyazawa, Tetsu Akimoto, Kazuyuki Shimada, Daisuke Nagata

**Affiliations:** ^1^Division of Nephrology, Department of Internal Medicine, Jichi Medical University, Shimotsuke, Tochigi, Japan; ^2^Department of Nephrology, Shin-Oyama City Hospital, Oyama, Tochigi, Japan; ^3^Department of Internal Medicine, Nasu Minami Hospital, Nasukarasuyama, Tochigi, Japan; ^4^Department of Cardiology, Shin-Oyama City Hospital, Oyama, Tochigi, Japan

**Keywords:** SGLT2 inhibition, body fluid homeostasis, vasopressin, copeptin, loop diuretic, renin-angiotensin aldosterone system, sympathetic nervous system

## Abstract

Sodium glucose cotransporter 2 (SGLT2) inhibitors have both glucose-lowering and diuretic effects. We recently reported that the SGLT2 inhibitor dapagliflozin exerts short-term fluid homeostatic action in patients with chronic kidney disease (CKD). However, the long-term effects of SGLT2 inhibitors on body fluid status in patients with CKD remain unclear. This was a prospective, non-randomized, open-label study that included a dapagliflozin treatment group (*n* = 73) and a control group (*n* = 24) who were followed for 6 months. Body fluid volume was measured using a bioimpedance analysis device. The extracellular water-to-total body water ratio (ECW/TBW), a predictor of renal outcomes, was used as a parameter for body fluid status (fluid retention, 0.400 ≤ ECW/TBW). Six-month treatment with dapagliflozin significantly decreased ECW/TBW compared with the control group (−0.65% ± 2.03% vs. 0.97% ± 2.49%, *p* = 0.0018). Furthermore, dapagliflozin decreased the ECW/TBW in patients with baseline fluid retention, but not in patients without baseline fluid retention (−1.47% ± 1.93% vs. −0.01% ± 1.88%, *p* = 0.0017). Vasopressin surrogate marker copeptin levels were similar between the control and dapagliflozin groups at 6 months (32.3 ± 33.4 vs. 30.6 ± 30.1 pmol/L, *p* = 0.8227). However, dapagliflozin significantly increased the change in copeptin levels at 1 week (39.0% ± 41.6%, *p* = 0.0010), suggesting a compensatory increase in vasopressin secretion to prevent hypovolemia. Renin and aldosterone levels were similar between the control and dapagliflozin groups at 6 months, while epinephrine and norepinephrine (markers of sympathetic nervous system activity) were significantly lower in the dapagliflozin group than in the control group. In conclusion, the SGLT2 inhibitor dapagliflozin ameliorated fluid retention and maintained euvolemic fluid status in patients with CKD, suggesting that SGLT2 inhibitors exert sustained fluid homeostatic actions in patients with various fluid backgrounds.

Clinical trial registration: https://www.umin.ac.jp/ctr/, identifier [UMIN000048568].

## 1 Introduction

Sodium-glucose cotransporter 2 (SGLT2) is mainly located in the brush border membrane of the renal early proximal tubule and mediates glucose and sodium reabsorption in the kidney (1). SGLT2 inhibitors exert glucose-lowering and diuretic effects (1–5). Several large-scale clinical trials from 2015 (EMPA-REG OUTCOME, DECLARE–TIMI 58, CANVAS Program, and CREDENCE trials) evaluated the cardiorenal protective effects of SGLT2 inhibitors in patients with type 2 diabetes (6–9). In two recent clinical trials of patients with chronic kidney disease (CKD) with or without diabetes (DAPA-CKD and EMPA-KIDNEY trials), the SGLT2 inhibitors dapagliflozin and empagliflozin significantly decreased the combined risk of a sustained decline in the estimated glomerular filtration rate, end-stage renal disease, and death from renal or cardiovascular causes (10, 11). Furthermore, in clinical trials involving a wide range of heart failure patients, regardless of ejection fraction (DAPA-HF trials, EMPEROR-Reduced, EMPEROR-Preserved, and DELIVER), SGLT2 inhibitors dapagliflozin and empagliflozin were associated with a significantly decreased risk of cardiovascular death and heart failure hospitalization (12–15). Based on the findings of these clinical trials, the use of SGLT2 inhibitors has expanded to the treatment of CKD and heart failure patients, regardless of diabetes (16).

Various mechanisms have been proposed to explain the cardiorenal protective effects of SGLT2 inhibitors. Among them, attenuation of glomerular hyperfiltration mediated by tubuloglomerular feedback is the most promising mechanism for explaining favorable renal outcomes, especially in patients with diabetes mellitus (1). In addition, recent animal studies have shown that the elevation of ketone bodies by SGLT2 inhibitors acts as a fuel for cardiorenal protection in diabetes mellitus (17, 18). Furthermore, the synthesis of erythropoietin and an increase in hemoglobin by SGLT2 inhibitors may reduce heart failure or renal events (17). However, thus far, the common mechanisms of SGLT2 inhibitors for explaining both kidney and heart protection in a broad range of patients with CKD and heart failure, regardless of diabetes, have remained unclear (17).

We recently showed that SGLT2 inhibitors can ensure a suitable fluid status, which may contribute to cardiorenal protection (16, 19–22). Similar to conventional diuretics, SGLT2 inhibitors exert mild natriuretic and glucosuria-induced osmotic diuretic properties (3, 19, 21, 23, 24). However, unlike loop diuretics, SGLT2 inhibitors ameliorate hypervolomia but have a low tendency to induce hypovolemia (3, 19–22, 25). In particular, in our clinical study of CKD patients with type 2 diabetes, treatment with dapagliflozin for 1 week decreased the fluid volume in patients with fluid retention but did not decrease the fluid volume in patients without fluid retention (22). The fluid homeostatic action of SGLT2 inhibitors is due to (1) a compensatory increase in fluid and food intake (19, 21) and (2) the suppression of excessive urine volume by vasopressin-induced solute-free water reabsorption in the renal collecting duct (19–21, 25, 26). Because both hypervolemia and hypovolemia are risk factors for the progression of renal and cardiovascular diseases (27–30), fluid homeostatic action of SGLT2 inhibitors may play a critical role in cardio-renal protection.

However, the sustained effects of SGLT2 inhibitors on body fluid status and hemodynamic parameters in CKD patients have not yet been evaluated. Therefore, we examined the effects of the SGLT2 inhibitor dapagliflozin on fluid status and hemodynamic parameters, including the antidiuretic hormone vasopressin and renin-angiotensin aldosterone system (RAAS), for 6 months in CKD patients with or without fluid retention.

## 2 Methods

### 2.1 Patients

This prospective, non-randomized, open-label study analyzed 97 patients with CKD at the Shin-Oyama City Hospital (Oyama, Tochigi, Japan) and Nasu Minami Hospital (Nasukarasuyama, Tochigi, Japan) between February 2016 and August 2022. At the time of entry, 101 patients had received dapagliflozin (dapagliflozin group), while 29 patients had not received dapagliflozin (the control group; Supplementary Figure S1). In the dapagliflozin group, 28 patients were excluded from the final analysis for the following reasons:12 who were lost to follow-up, 5 with incomplete bioimpedance analysis (BIA) data, 4 who started dialysis, 3 who discontinued dapagliflozin (1 for appetite loss, 1 for skin pruritus, 1 for refusal of medication), 3 who died (2 with heart failure, 1 in a traffic accident), and 1 due to lack of a stored serum sample. In the control group, 5 patients were excluded from the final analysis due to the following reasons:3 who were lost to follow-up, 1 who started dialysis, and 1 who started dapagliflozin. Finally, 73 patients received dapagliflozin (5 mg, *n* = 15, 10 mg, *n* = 58), whereas 24 patients did not receive dapagliflozin as a control group. The entry criteria were as follows: (1) an estimated glomerular filtration rate (eGFR) of >15 but <59 mL/min/1.73 m^2^ with or without proteinuria or (2) eGFR >60 mL/min/1.73 m^2^ with proteinuria. Treatment drug (use or non-use of dapagliflozin) was determined according to the judgment of the attending physicians. The main indication for dapagliflozin administration is diabetes or chronic glomerular nephropathy, as dapagliflozin induces favorable renal outcomes in these patients (10, 31). Drug selection primarily depended on the cause of CKD, which reduced the possibility of bias. The exclusion criteria were a history of renal replacement therapy, current dialysis, type 1 diabetes, active malignancy, and implanted pacemakers. The observation period after administration was 6 months. Diuretics were not discontinued at the time of entry and dose changes in concomitant medications were made at the discretion of the attending physician during the study period.

This study was registered in the University Hospital Medical Information Network Clinical Trials Registry System (UMIN-CTR) as the DAPA-BODY Trial (UMIN000048568) and was conducted in accordance with the ethical principles of the Declaration of Helsinki. The study protocol was approved by the independent ethics committees of Shin-Oyama City Hospital (approval number: SOR2020-004) and Nasu Minami Hospital (approval number:2016–03). Written informed consent to participate in this study was obtained from all patients.

### 2.2 Study endpoints

The primary endpoint of this trial was the change in the extracellular water (ECW) to total body water (TBW) ratio using the BIA device for 6 months. The secondary endpoints were changes in estimated plasma volume and hemodynamic parameters, including copeptin, renin, and aldosterone, after 6 months.

### 2.3 Blood and urine analyses

Blood and urine samples were collected at baseline and after 6 months. The eGFR was calculated using the Modification of Diet in Renal Disease study coefficients modified for the Japanese population (32). Changes in the estimated plasma volume were calculated using the Strauss formula as follows: Hb baseline/Hb 6 months × [(100 – Ht 6 months) / (100 – Ht baseline) – 1] × 100 (33). The Strauss formula was used as a proxy to assess the traditional human and rodent plasma volume measurements with ^125^I-human serum albumin (20, 34, 35). Serum osmolarity was calculated using the following formula: 2 × serum Na^+^ (mEq/L) + plasma glucose (mg/dL) / 18 + blood urea nitrogen (mg/dL) / 2.8 (4, 36). Serum copeptin, a surrogate marker of vasopressin, was measured by Thermo Fisher Scientific using an automated immunoluminometric assay (ultra-sensitive B•R•A•H•M•S Copeptin proAVP; Thermo Fisher Scientific, Hennigsdorf, Germany) (37, 38). Serum samples were analyzed using enzyme-linked immunosorbent assay (ELISA) according to the manufacturer’s instructions: renin ELISA kit (DRG Instruments GmbH, Frauenbergstr, Marburg, Germany) (39) and aldosterone ELISA kit (IBL Co., Ltd., Fujioka, Japan) (40). Plasma epinephrine and norepinephrine were measured in the SRL laboratory (Hachioji, Tokyo, Japan) (41).

### 2.4 Measurement of the fluid volume using a BIA device

Body fluid volume was measured using a BIA device with eight tactile electrodes (InBody S10; InBody Japan Inc., Tokyo, Japan) before and 6 months after entry, similar to our previous studies (3, 4, 22, 41). The intracellular water (ICW), ECW, TBW (ICW + ECW), ECW-to-TBW ratio (ECW/TBW), fat mass, and bone mineral content were calculated from the sum of each segment using equations in the BIA software program. As ECW/TBW is a marker of extracellular fluid status and predictor of renal outcome (42), we adopted it as a marker of body fluid status. Based on the InBody S10 user manual (43), patients were classified into the following subgroups: fluid retention (−), ECW/TBW <0.400, and fluid retention (+), ECW/TBW ≥0.400.

### 2.5 Statistical analyses

The data are expressed as mean (standard deviation). Paired or unpaired *t*-tests were used to compare two variables, as appropriate. The normality of the data distribution was determined using the Shapiro–Wilk test. Non-normally distributed data are presented as medians and interquartile ranges, and Wilcoxon’s rank sum test was used for between-group comparisons. Correlations between variables were analyzed using Pearson’s correlation test. Repeated measures analysis of variance was performed to identify statistically significant differences in copeptin measurements among the three time points (baseline, 1 week, and 6 months). Patients without complete BIA data at baseline and 6 months were excluded from the final analysis. The JMP 14.3.0 statistical software program (SAS Institute, Inc., Cary, NC, United States) was used for statistical analyses. Statistical significance was set at *p* < 0.05.

## 3 Results

### 3.1 Patients’ characteristics

Of the 97 enrolled patients with CKD, 73 were assigned to the dapagliflozin group and 24 were assigned to the control group. Diastolic blood pressure, uric acid, and renin levels were significantly higher in the control group than in the dapagliflozin group (Table 1). In contrast, the prevalence of diabetes, plasma glucose, and HbA1c levels was significantly higher in the dapagliflozin group than in the control group (Table 1).

**Table 1:** Baseline characteristics of the control and dapagliflozin-treatment groups.

Characteristics	Control (*n* = 24)	Dapagliflozin (*n* = 73)	*p* value
Age, years	72.0 [57.8–79.8]	68.0 [59.5–73.5]	0.155
Male gender, *n* (%)	20 (83.3)	47 (64.4)	0.070
Body weight, kg	66.9 (10.9)	68.9 (17.2)	0.606
BMI, kg/m^2^	25.0 (3.8)	27.0 (4.7)	0.055
Diabetes, *n* (%)	2 (8.3)	42 (57.5)	<0.0001
Systolic BP, mmHg	144 (21)	136 (19)	0.084
Diastolic BP, mmHg	82 (13)	75 (11)	0.037
Heart rate, beats/min	83 (10)	78 (12)	0.099
Hemoglobin, g/dL	13.2 (2.2)	12.6 (2.0)	0.224
Hematocrit, %	39.8 (6.1)	37.9 (5.5)	0.166
Plasma glucose, mg/dL	108 [103–150]	138 [115–173]	0.024
HbA1c, %	6.0 (0.6)	7.2 (0.9)	<0.0001
BNP, pg./mL	16.3 [3.8–52.7]	22.4 [13.1–85.6]	0.133
Serum albumin, g/dL	3.9 (0.4)	3.9 (0.6)	0.640
BUN, mg/dL	25.3 [20.8–39.1]	25.6 [18.5–38.4]	0.603
Serum creatinine, mg/dL	1.69 [1.37–2.97]	1.69 [1.17–2.29]	0.116
eGFR, mL/min/1.73 m^2^	24.6 [17.0–39.0]	31.3 [20.6–47.5]	0.077
Uric acid, mg/dL	6.8 (1.3)	6.0 (1.3)	0.011
Serum Na^+^, mEq/L	139.6 (1.8)	140.5 (2.8)	0.125
Serum K^+^, mEq/L	4.5 (0.7)	4.4 (0.8)	0.553
Copeptin, pmol/L	17.0 [10.5–33.9]	21.0 [9.0–35.1]	0.574
Renin, pg./mL	75.9 [20.7–113.0]	24.9 [1.9–74.4]	0.021
Aldosterone, pg./mL	320 (252)	277 (211)	0.445
Proteinuria, g/gCr	0.52 [0.16–3.30]	1.07 [0.32–3.52]	0.504
ICW, L	21.0 (3.4)	21.0 (4.7)	0.984
ECW, L	13.7 (2.0)	13.9 (3.0)	0.771
TBW, L	34.7 (5.3)	34.9 (0.8)	0.921
ECW/TBW	0.395 (0.012)	0.399 (0.015)	0.324
Fat mass, kg	20.1 (7.2)	22.8 (9.4)	0.198
Bone mineral content, kg	2.5 (0.4)	2.5 (0.6)	0.932
Concomitant diuretics, %	29.2	31.5	0.830
Loop diuretics, %	8.3	26.0	0.068
Thiazide diuretics, %	4.2	4.1	0.990
MR antagonist, %	8.3	6.9	0.807
Tolvaptan, %	4.2	4.1	0.990
ACE inhibitor or ARB, %	58.3	61.1	0.810
Calcium channel blocker, %	79.2	75.0	0.679

Data are presented as mean (standard deviation) or median [interquartile range], as appropriate. BMI, body mass index; BP, blood pressure; BNP, brain natriuretic peptide; BUN, blood urea nitrogen; eGFR, estimated glomerular filtration rate; ICW, intracellular water; ECW, extracellular water; TBW, total body water; MR, mineralocorticoid receptor; ACE, angiotensin-converting enzyme; ARB, angiotensin receptor blocker.

Dapagliflozin-treated patients were further divided into fluid retention (+) and fluid retention (−) groups. Body weight, body mass index, prevalence of diabetes, hemoglobin, hematocrit, serum albumin, eGFR, ICW, and fat mass were significantly higher in the fluid retention (−) group than in the fluid retention (+) group (Table 2). In contrast, age, brain natriuretic peptide (BNP), blood urea nitrogen, serum creatinine, urine proteinuria, ECW/TBW, and percentages of concomitant diuretic use (loop diuretic and thiazine), tolvaptan, and calcium channel blocker were significantly higher in the fluid retention (+) group than in the fluid retention (−) group (Table 2).

**Table 2:** Baseline characteristics of dapagliflozin-treated patients with and without fluid retention.

Characteristics	Fluid retention (−) (*n* = 41)	Fluid retention (+) (*n* = 32)	*p* value
Age, years	61.9 (13.1)	70.8 (11.2)	0.003
Male gender, *n* (%)	25 (61.0)	22 (68.8)	0.491
Body weight, kg	71.0 [63.9–84.6]	65.0 [50.3–72.0]	0.002
BMI, kg/m^2^	28.4 (5.0)	25.3 (3.5)	0.004
Diabetes, *n* (%)	23 (56.1)	8 (25.0)	0.008
Systolic BP, mmHg	133 (18)	140 (20)	0.105
Diastolic BP, mmHg	77 (11)	73 (11)	0.156
Heart rate, beats/min	76 (11)	79 (13)	0.371
Hemoglobin, g/dL	13.6 (1.8)	11.4 (1.5)	<0.0001
Hematocrit, %	40.6 (4.9)	34.7 (4.4)	<0.0001
Plasma glucose, mg/dL	128 [107–152]	158 [126–183]	0.145
HbA1c, %	7.2 (0.7)	7.2 (1.2)	0.897
BNP, pg./mL	18.6 [8.5–30.8]	72.5 [20.3–409.2]	0.002
Serum albumin, g/dL	4.1 (0.4)	3.6 (0.6)	<0.0001
BUN, mg/dL	24.3 (2.1)	35.6 (2.4)	0.0007
Serum creatinine, mg/dL	1.62 (0.86)	2.12 (0.91)	0.018
eGFR, mL/min/1.73 m^2^	40.7 (19.0)	27.5 (12.8)	0.001
Uric acid, mg/dL	5.9 (1.2)	6.1 (1.4)	0.524
Serum Na^+^, mEq/L	141 (2)	140 (4)	0.741
Serum K^+^, mEq/L	4.3 (0.9)	4.5 (0.6)	0.267
Copeptin, pmol/L	15.2 [8.2–30.3]	22.9 [9.7–44.2]	0.186
Renin, pg./mL	30.3 [9.9–110.9]	36.3 [21.5–93.0]	0.796
Aldosterone, pg./mL	326 [141–488]	257 [163–590]	0.822
Proteinuria, g/gCr	0.80 [0.28–2.30]	1.73 [0.59–3.99]	0.037
ICW, L	22.1 (4.6)	19.6 (4.6)	0.030
ECW, L	14.0 (2.9)	13.8 (3.1)	0.731
TBW, L	36.1 (7.5)	33.4 (7.6)	0.143
ECW/TBW	0.390 [0.384–0.394]	0.409 [0.402–0.416]	<0.0001
Fat mass, kg	24.3 [18.9–31.7]	19.9 [14.6–23.9]	0.015
Bone mineral content, kg	2.5 [2.2–2.8]	2.4 [2.0–2.7]	0.198
Concomitant diuretics, %	7.3	62.5	<0.0001
Loop diuretics, %	7.3	50.0	<0.0001
Thiazide diuretics, %	0.0	9.4	0.045
MR antagonist, %	4.9	9.4	0.450
Tolvaptan, %	0.0	9.4	0.045
ACE inhibitor or ARB, %	63.4	58.1	0.645
Calcium channel blocker, %	65.9	87.1	0.039

Fluid retention (−), ratio of extracellular water (ECW) to total body water (TBW; ECW/TBW) < 0.400; fluid retention (+), ECW/TBW ≥ 0.400. Data are presented as mean (standard deviation) or median [interquartile range], as appropriate. BMI, body mass index; BP, blood pressure; BNP, brain natriuretic peptide; BUN, blood urea nitrogen; eGFR, estimated glomerular filtration rate; ICW, intracellular water; MR, mineralocorticoid receptor; ACE, angiotensin-converting enzyme; ARB, angiotensin-receptor blocker.

### 3.2 Changes in body fluid status

**Figure 1:**
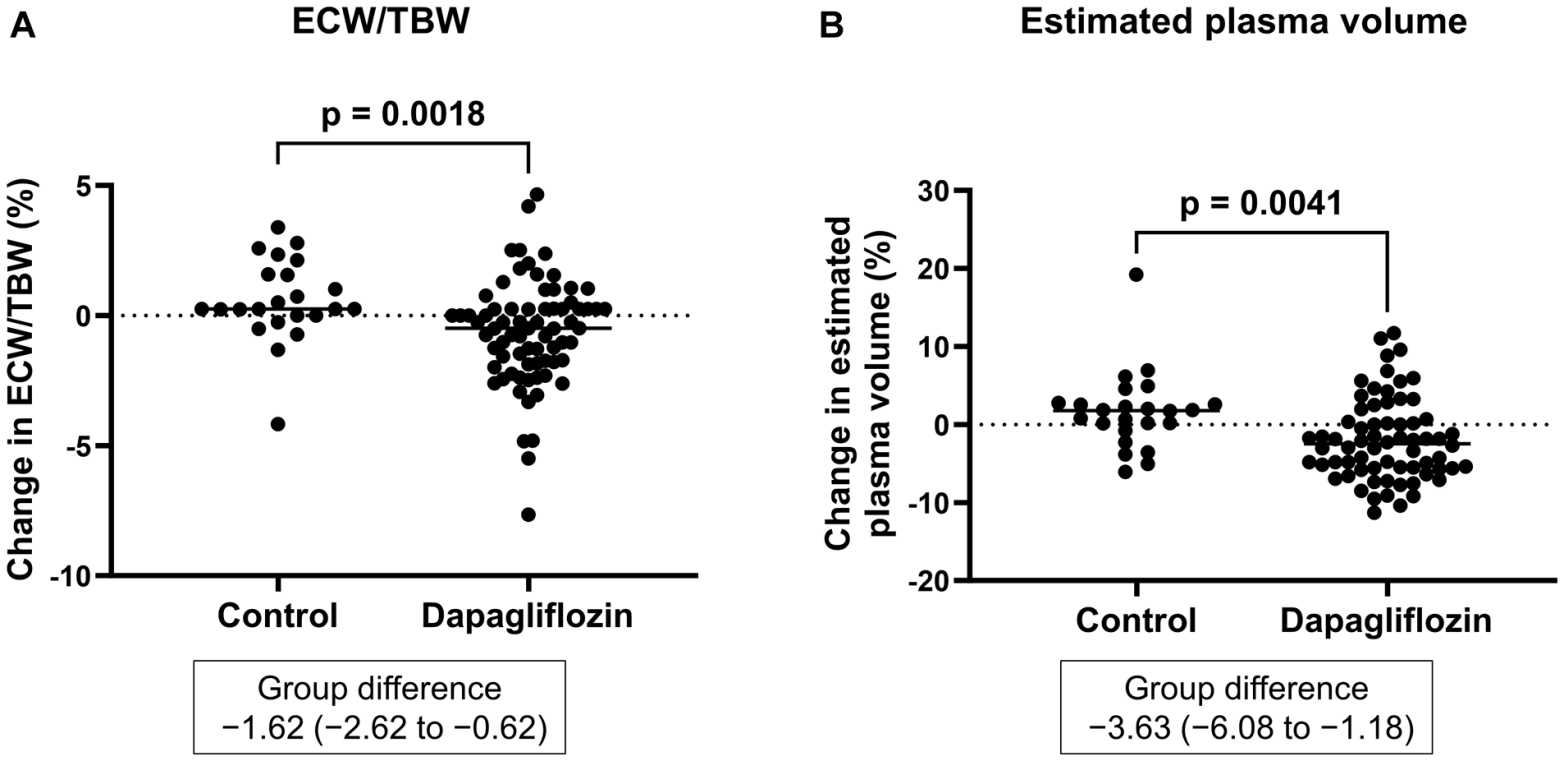
**(A)** Comparative changes in the extracellular water-to-total body water ratio (ECW/TBW) at 6 months between the control and dapagliflozin group. **(B)** Comparative percent changes in the estimated plasma volume at 6 months between the control and dapagliflozin groups.

**Figure 2:**
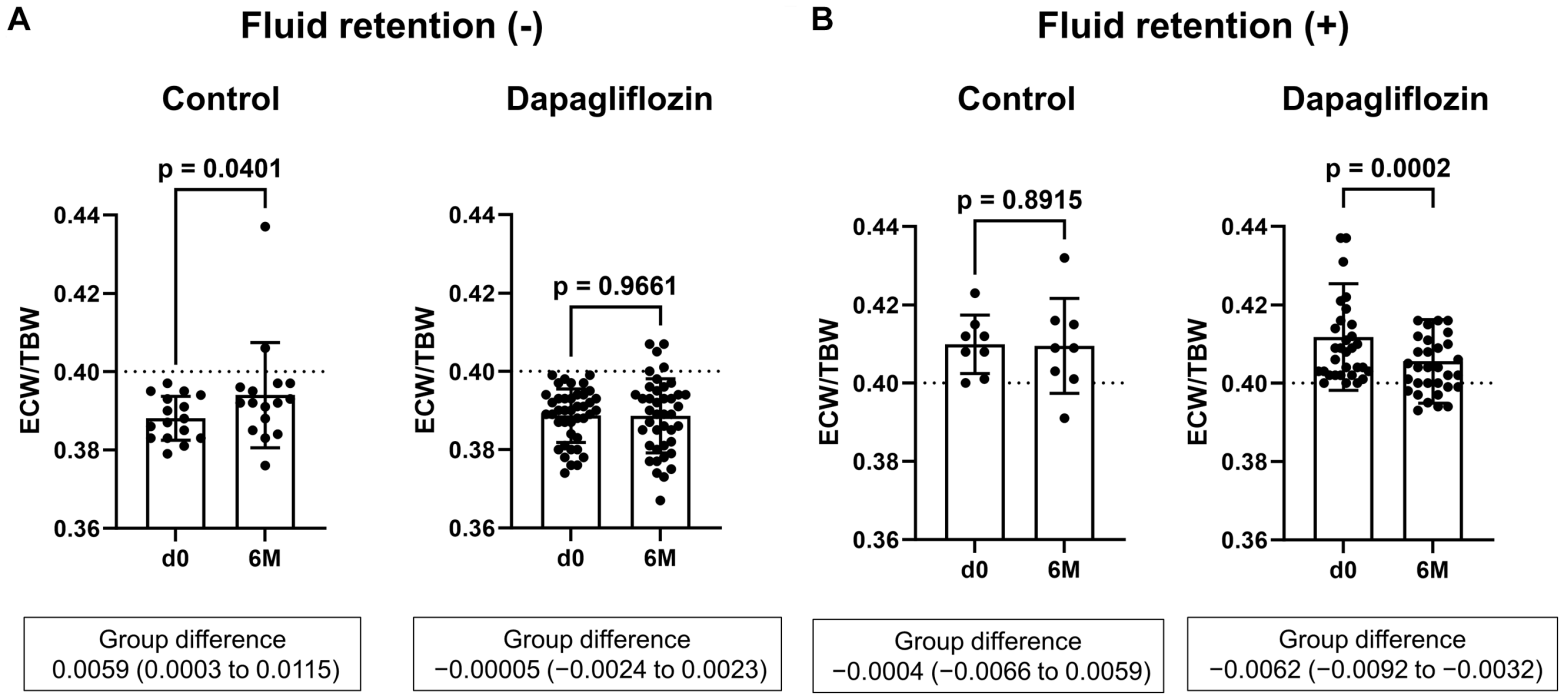
Comparative absolute changes in the extracellular water-to-total body water ratio (ECW/TBW) from baseline to 6 months between the control and dapagliflozin groups. **(A)** Patients without baseline fluid retention (ECW/TBW <0.400) and **(B)** patients with baseline fluid retention (ECW/TBW ≥0.400). d0, baseline; 6 M, 6 months after entry.

**Figure 3:**
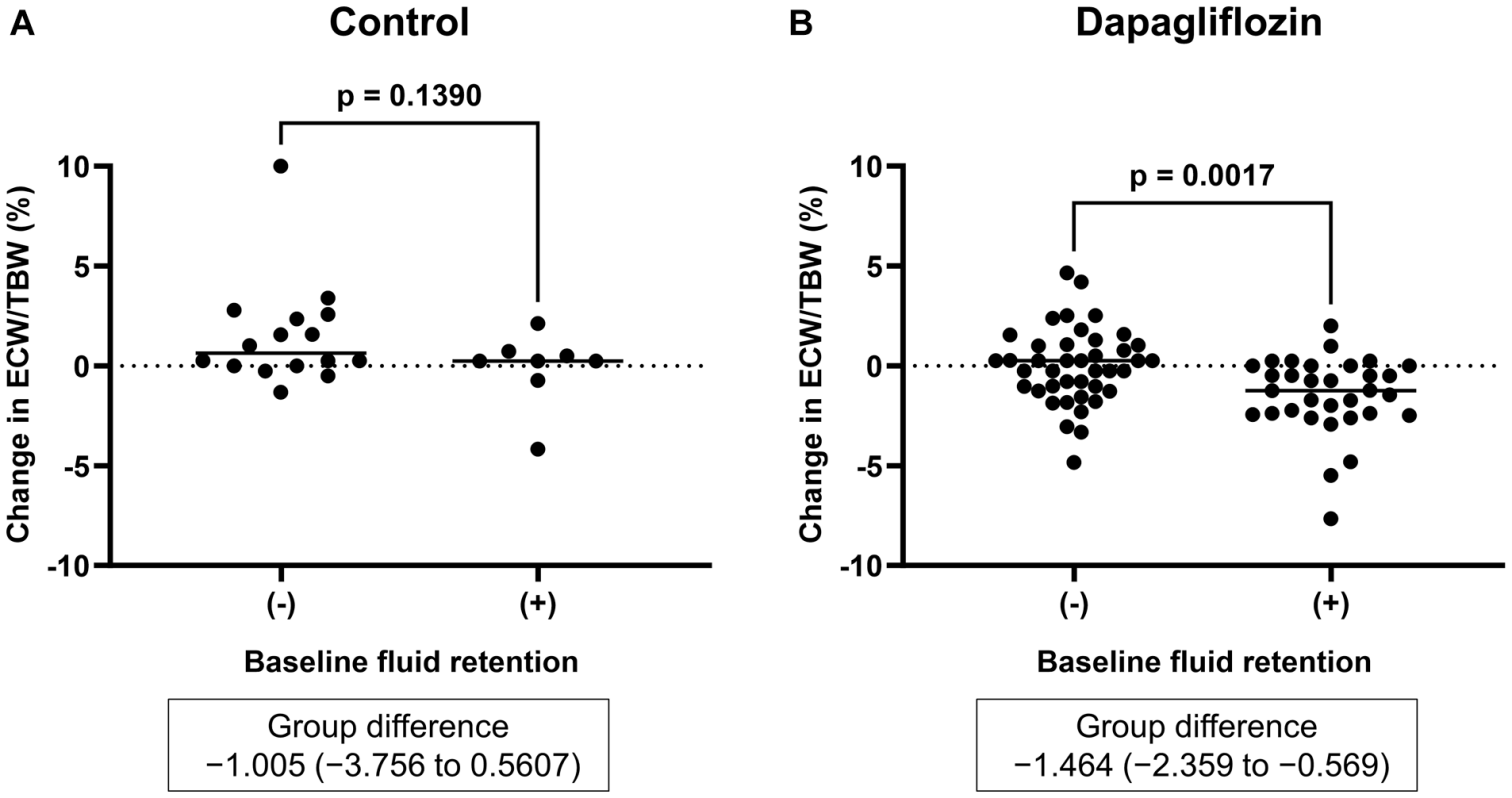
Comparative changes in the extracellular water-to-total body water ratio (ECW/TBW) at 6 months between patients without and with baseline fluid retention (ECW/TBW ≥0.400). **(A)** Control group and **(B)** dapagliflozin group.

**Figure 4:**
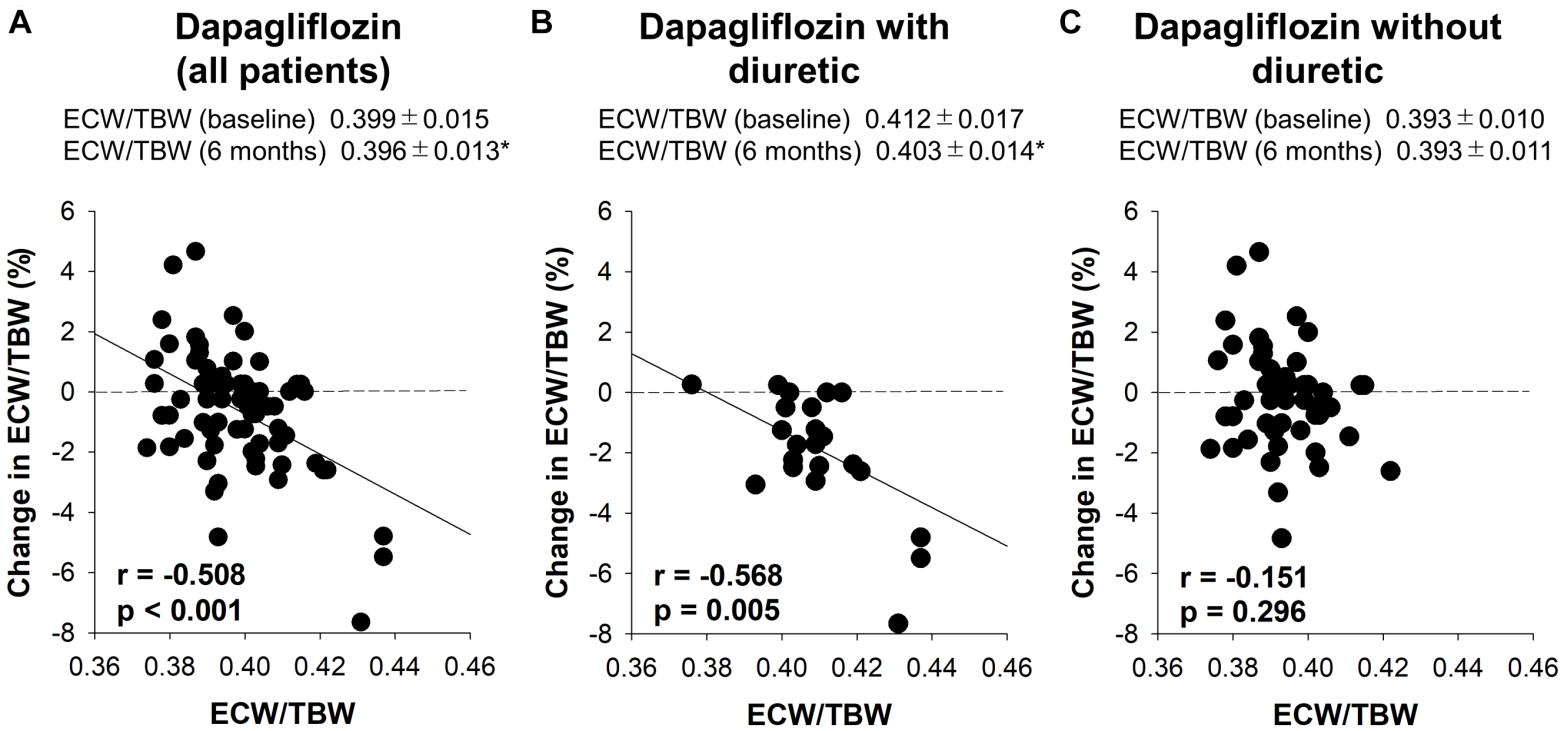
The correlation between the baseline extracellular water-to-total body water ratio (ECW/TBW) and the change in the ECW/TBW at 6 months. **(A)** Dapagliflozin (all patients), **(B)** dapagliflozin combined with a diuretic, and **(C)** dapagliflozin without a diuretic. *p < 0.05 vs. baseline.

Six-month treatment with dapagliflozin significantly decreased ECW/TBW compared with the control group (−0.65 ± 2.03% vs. 0.97 ± 2.49%, *p* = 0.0018). Six-month treatment with dapagliflozin significantly decreased the ECW/TBW in patients with baseline fluid retention (−1.47 ± 1.93% vs. -0.01 ± 1.88%, *p* = 0.0017) and estimated plasma volume (−1.97% ± 5.26% vs. 1.66% ± 4.98%, *p* = 0.0041) compared with the control group (Figure 1). Furthermore, in patients without fluid retention, ECW/TBW was significantly increased in the control group, but not in the dapagliflozin-treated group (Figure 2). In patients with fluid retention, ECW/TBW did not significantly change in the control group; however, dapagliflozin significantly decreased ECW/TBW at 6 months (Figure 2). In the control group, there was no significant difference in the changes in ECW/TBW between patients with and without fluid retention at 6 months (1.50% ± 2.63% vs. −0.10% ± 1.82%, *p* = 0.1390; Figure 3). In contrast, in the dapagliflozin group, the change in the ECW/TBW in patients with fluid retention was negative and was significantly higher than that in patients without fluid retention (−0.01% ± 1.88% vs. −1.47% ± 1.93%, *p* = 0.0017; Figure 3). In the dapagliflozin group, there was a significant negative correlation between baseline ECW/TBW and change in ECW/TBW at 6 months (*r =* −0.508, *p* < 0.001; Figure 4). Furthermore, in patients treated with dapagliflozin and diuretics (loop diuretics and/or thiazide), there was a significant correlation between baseline ECW/TBW and the change in ECW/TBW (*r* = −0.568, *p* = 0.005; Figure 4). In contrast, in patients treated with dapagliflozin without diuretics, there was no significant correlation between baseline ECW/TBW and the change in ECW/TBW (*r* = −0.151, *p* = 0.296; Figure 4).

### 3.3 Hemodynamic and sympathetic nervous system parameters

**Figure 5:**
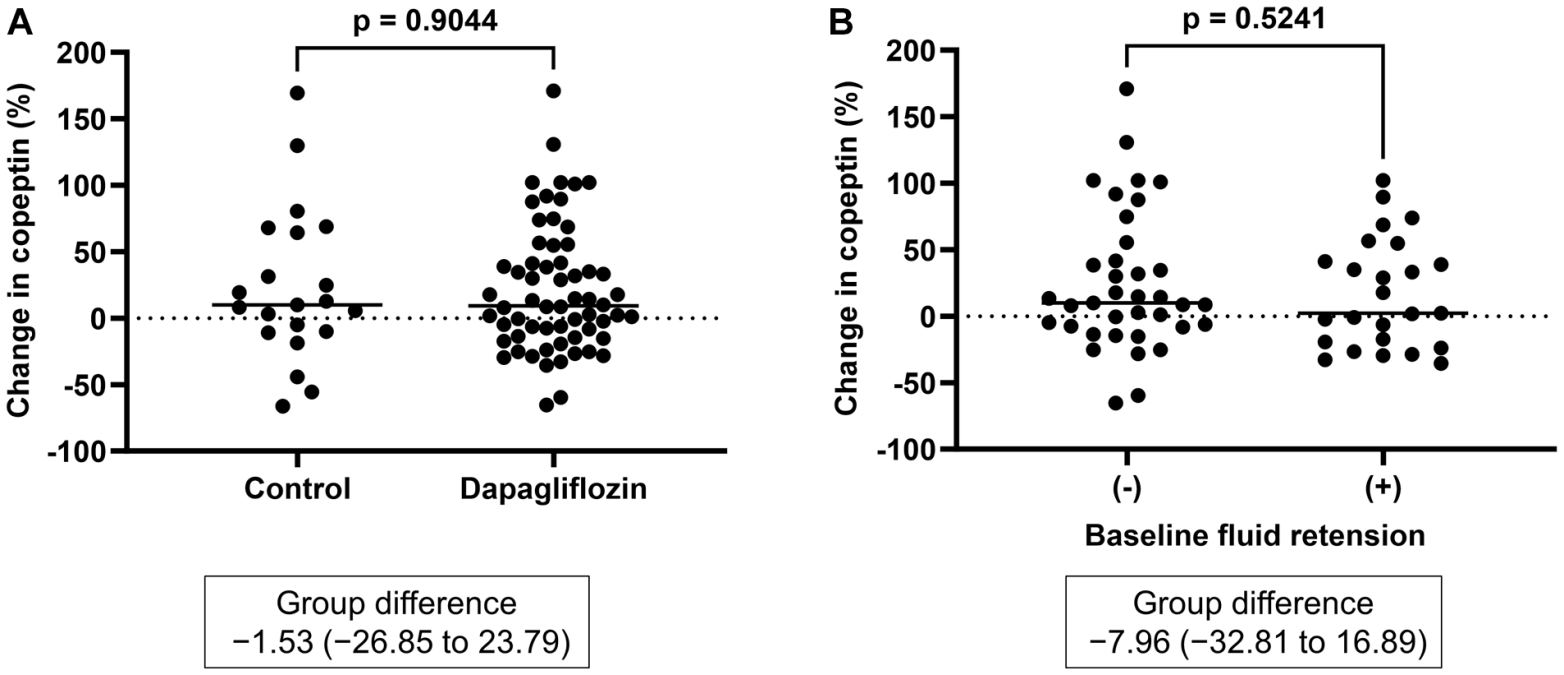
**(A)** Comparative changes in copeptin levels at 6 months between the control and dapagliflozin groups. **(B)** Comparative changes in copeptin levels at 6 months between patients without and with baseline fluid retention.

**Figure 6:**
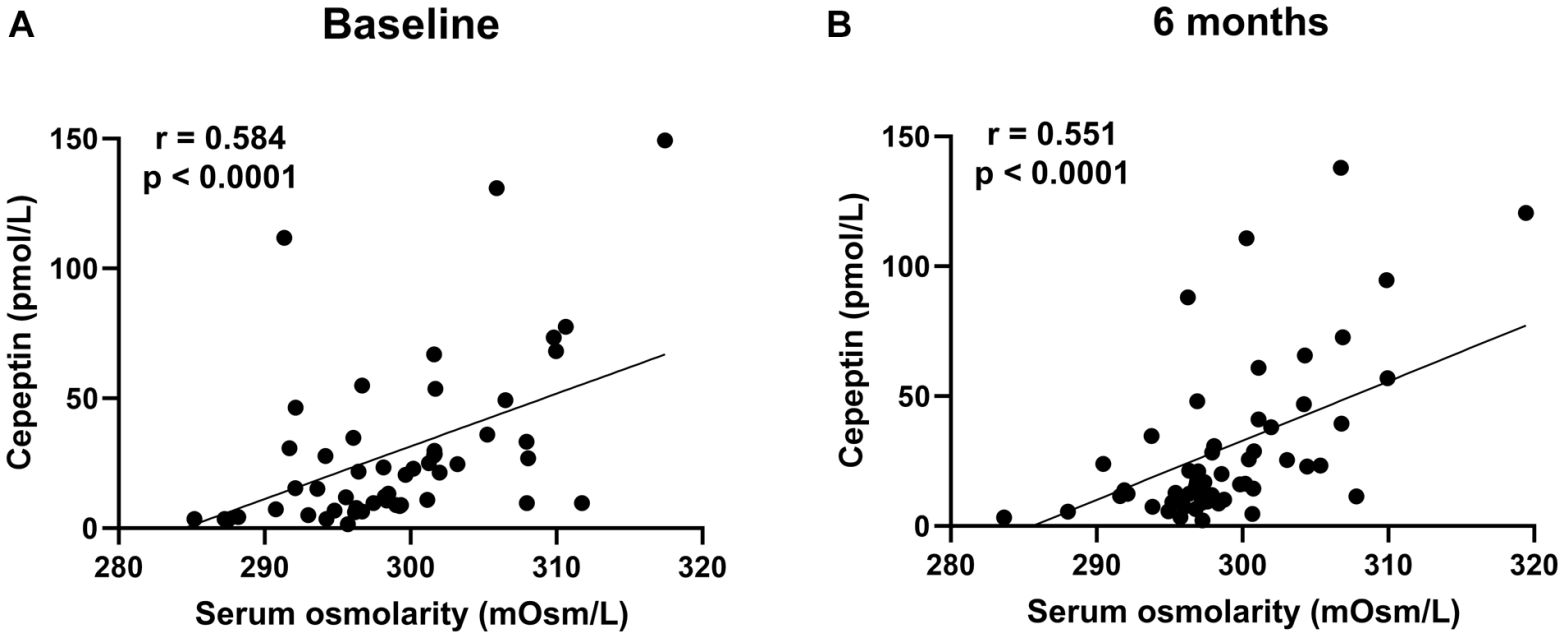
The correlation between serum osmolarity and copeptin levels in patients treated with dapagliflozin at baseline **(A)** and 6 months **(B)**.

There was no significant difference in the absolute value (32.3 ± 33.4 vs. 30.6 ± 30.1 pmol/L, *p =* 0.8227) and the change in copeptin between the control and dapagliflozin groups at 6 months (Figure 5). Furthermore, in the dapagliflozin group, there was no significant difference in the change in copeptin levels between the patients with and without fluid retention at 6 months (Figure 5). In addition, copeptin levels were positively and significantly correlated with serum osmolality both at baseline (*r =* 0.584, *p* < 0.0001) and at 6 months (*r* = 0.551, *p* < 0.0001; Figure 6).

**Figure 7:**
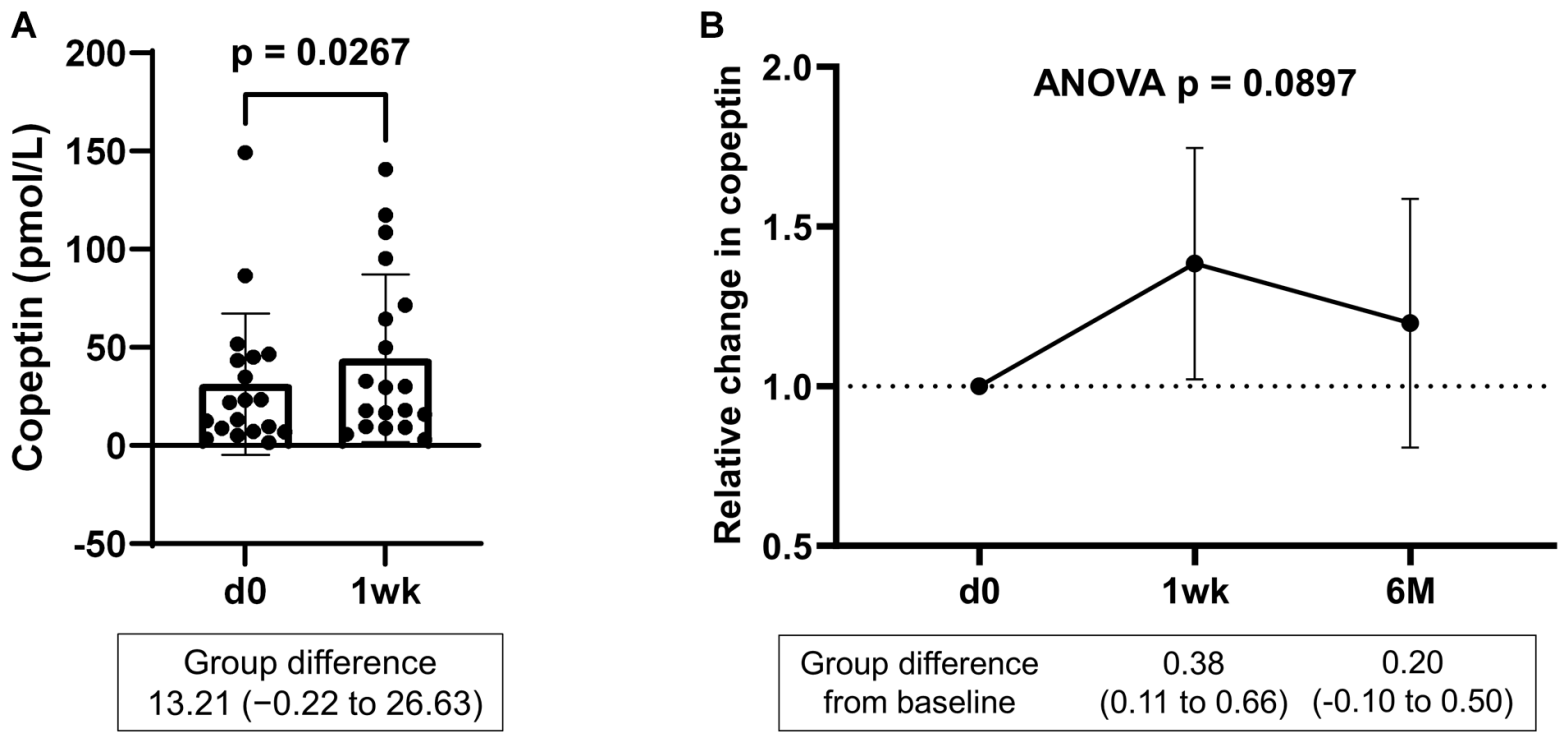
**(A)** Copeptin levels in patients treated with dapagliflozin at baseline and 1 week (*n* = 19). **(B)** Relative change in copeptin levels in patients treated with dapagliflozin at 1 week and 6 months (*n* = 10). d0; baseline, 1wk; 1 week, 6 M; 6 months after administration.

**Figure 8:**
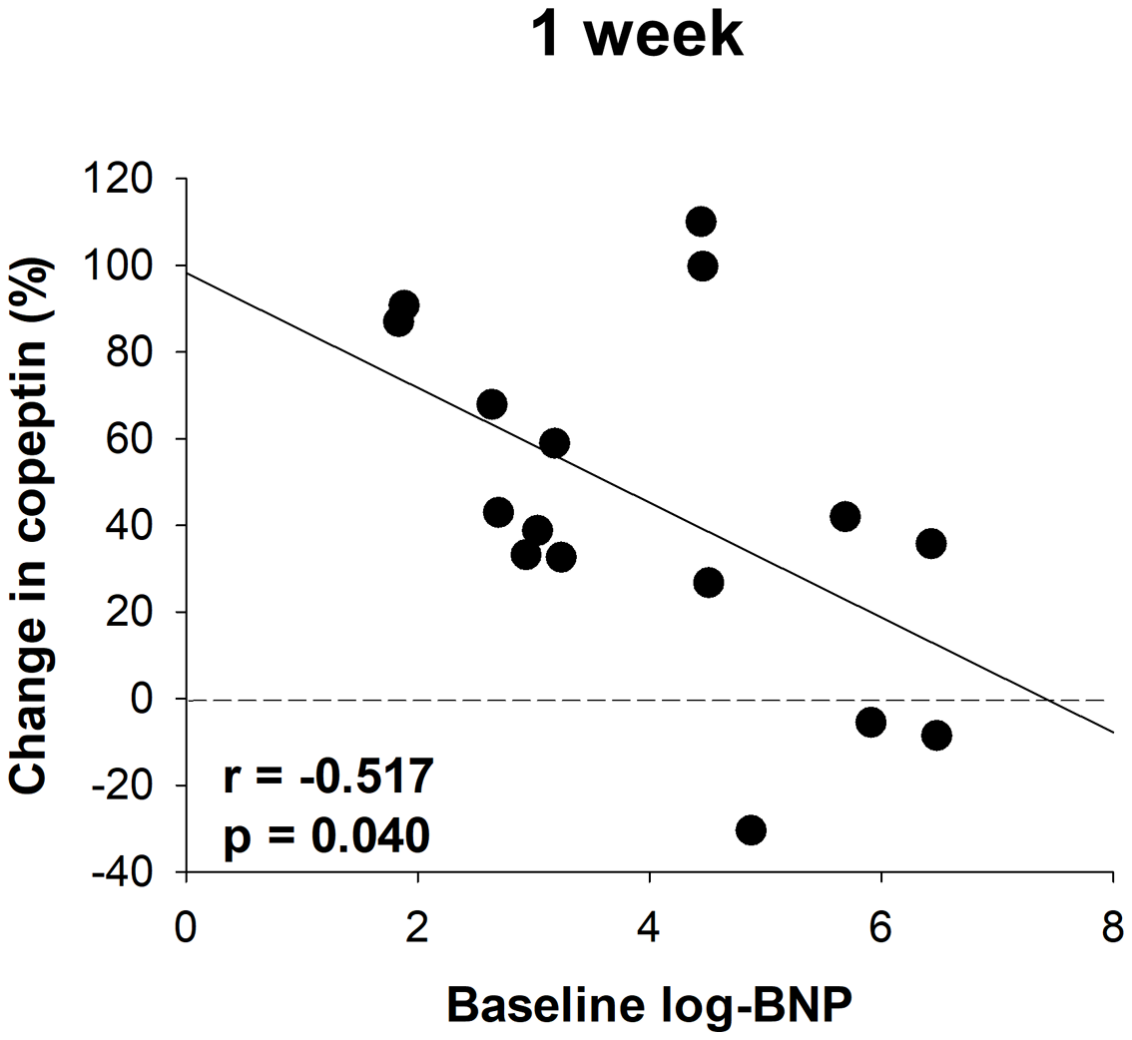
The correlation between log-BNP and the change in copeptin levels in patients treated with dapagliflozin at 1 week (*n* = 16).

Serum samples were collected from a portion of the dapagliflozin group after 1 week. Copeptin levels 1 week after dapagliflozin initiation were significantly higher than those at baseline (*n* = 19; Figure 7). Similarly, dapagliflozin significantly increased the change in copeptin levels at 1 week (39.0 ± 41.6%, *p* = 0.0010). Furthermore, in patients with stored samples at three time points (*n* = 10), we measured the relative changes in copeptin levels at 1 week and 6 months with respect to the baseline level. Copeptin levels increased at 1 week, but returned to their initial values at 6 months (Figure 7). In addition, in dapagliflozin-treated patients, log-BNP at baseline was negatively and significantly correlated with the change in copeptin levels at 1 week (*n* = 16, *r* = 0.551, *p* < 0.0001; Figure 8).

The serum renin and aldosterone levels were measured. At 6 months, the levels of renin (control 56.9 ± 42.0 vs. dapagliflozin 63.7 ± 43.6 pg./mL, *p* = 0.568) and aldosterone (control 419 ± 225 vs. dapagliflozin 370 ± 190 pg./mL; *p* = 0.385) were similar between the control and dapagliflozin groups. Likewise, the percent changes in renin (control −21.9% ± 74.1% vs. dapagliflozin 16.4% ± 92.8%, *p* = 0.138) and aldosterone (control −0.7% ± 27.5% vs. dapagliflozin 59.2% ± 162.6%, *p* = 0.163) at 6 months were not significantly different between the control and dapagliflozin groups. In the dapagliflozin group, the levels of renin (*n* = 14, baseline 48.1 ± 37.1 vs. 1 week 59.2 ± 162.6 pg./mL, *p* = 0.625) and aldosterone (*n* = 8, baseline 348 ± 199 vs. 1 week 364 ± 212 pg./mL, *p* = 0.612) at 1 week were similar to the baseline levels.

**Figure 9:**
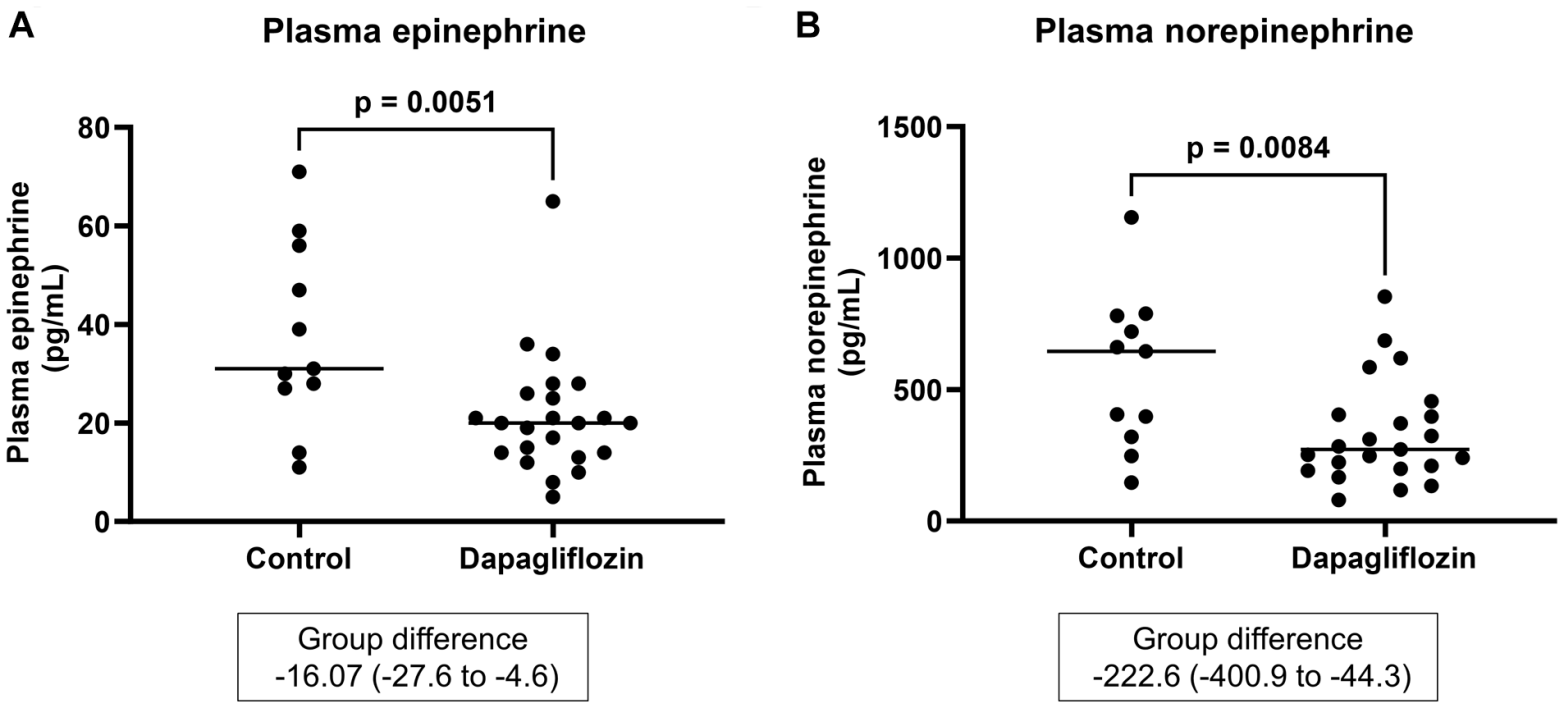
Comparative concentrations of plasma epinephrine **(A)** and norepinephrine **(B)** at 6 months between the control (*n* = 11) and dapagliflozin (*n* = 23) groups.

Plasma epinephrine and norepinephrine were measured as parameters of sympathetic nervous system activity. At 6 months, the plasma epinephrine and norepinephrine levels were significantly lower in the dapagliflozin group than in the control group (Figure 9).

## 4 Discussion

In this study, the SGLT2 inhibitor dapagliflozin decreased the extracellular fluid parameter ECW/TBW and estimated plasma volume for 6 months. Recent clinical studies in patients with type 2 diabetes with or without heart failure showed that the SGLT2 inhibitors dapagliflozin, empagliflozin, canagliflozin, and ipragliflozin significantly reduced the estimated extracellular volume and estimated plasma volume for up to 24 months (33, 44–46). Similar to these studies, the current study in CKD patients with and without diabetes showed that dapagliflozin significantly reduced fluid volume parameters, including ECW/TBW and estimated plasma volume.

A novel and important finding of this study was that the SGLT2 inhibitor dapagliflozin exerted a sustained fluid homeostatic action in patients with various fluid backgrounds. Specifically, dapagliflozin ameliorated fluid retention and maintained euvolemic fluid status for 6 months in patients with CKD, regardless of baseline fluid retention. A recent clinical study showed that the SGLT2 inhibitor empagliflozin did not alter euvolemic fluid status for 24 weeks (ECW/TBW: baseline 0.392 and 24 weeks 0.391, *p* = 0.460) in patients with acute myocardial infarction and type 2 diabetes mellitus (47). Another notable study in patients with type 2 diabetes with preserved renal function reported that dapagliflozin and empagliflozin transiently decreased ECW at 30 days but returned it to the baseline level at 180 days (48). These two clinical studies suggest that SGLT2 inhibitors may exert long-term fluid homeostatic actions in patients without fluid retention. However, whether SGLT2 inhibitors can normalize and maintain fluid volume status in patients with various fluid backgrounds has not yet been evaluated. The present study revealed that dapagliflozin ameliorates hypervolemia in patients with baseline fluid retention and maintains euvolemia in patients without baseline fluid retention for 6 months. In contrast, in the control group, the extracellular fluid parameter ECW/TBW significantly increased in patients without baseline fluid retention and did not change in patients with baseline fluid retention. These data suggest that the SGLT2 inhibitor dapagliflozin exerts long-term fluid homeostatic action, similar to our short-term (1 week) study in patients with and without baseline fluid retention (22).

Regulation of vasopressin secretion is the most promising mechanism for sustained fluid homeostasis by SGLT2 inhibitors. Several clinical studies in patients with diabetes and preserved kidney function showed that dapagliflozin and empagliflozin increased the plasma levels of copeptin, a marker of vasopressin secretion (26, 36, 49, 50). Similarly, in non-diabetic patients with CKD, dapagliflozin increased plasma copeptin levels over 2 weeks of treatment (51). In addition to these clinical data, we showed that the SGLT2 inhibitor ipragliflozin in diabetic and non-diabetic rats increased osmotic diuresis-induced vasopressin secretion and solute-free water reabsorption in the collecting duct (19, 20). Importantly, this compensatory water reabsorption induced by SGLT2 inhibitors helps maintain euvolemic fluid status in the long-term (19, 20).

The detailed data in this study emphasize the critical role of vasopressin in fluid homeostasis during SGLT2 inhibition. First, a positive correlation between serum osmolality and copeptin levels was confirmed both at baseline and 6 months after dapagliflozin administration, indicating that osmolality-dependent vasopressin secretion is continuously activated to maintain euvolemic fluid status. Second, copeptin levels were significantly increased in response to dapagliflozin at 1 week but subsequently decreased close to the baseline level by 6 months. Third, baseline BNP, a biomarker of volume status (28, 52), was negatively and significantly correlated with the change in copeptin levels after one-week treatment with dapagliflozin. These second and third results indicated that the diuretic action of dapagliflozin induces a transient and clear increase in vasopressin secretion to prevent hypovolemia, especially in patients without fluid retention. Taken together, these findings suggest that the regulation of vasopressin secretion during SGLT2 inhibition may play a critical role in ameliorating fluid retention and maintaining the euvolemic fluid status.

RAAS activation is involved in maintaining body fluid status (54, 55), but whether SGLT2 inhibitors activate RAAS is controversial (2). In patients with type 2 diabetes and preserved kidney function, dapagliflozin increases plasma renin levels at 2, 6, and 12 weeks (26, 50). Similarly, in non-diabetic patients with or without CKD, dapagliflozin and empagliflozin increased plasma renin activity and aldosterone from 4 to 6 weeks (52, 56). In contrast, another study showed that dapagliflozin and empagliflozin did not significantly increase plasma renin activity and aldosterone levels at 30 and 90 days in patients with type 2 diabetes (37). The reasons for the different changes in RAAS parameters caused by SGLT2 inhibitors have not been clearly evaluated. However, coordinated changes in the body fluid status and RAAS activity have been reported in several studies. Our recent animal study showed that the SGLT2 inhibitor ipragliflozin did not alter renin activity, plasma volume, and body fluid volume, whereas the loop diuretic furosemide increased renin activity accompanied by a reduction in plasma volume and body fluid volume (20). Schork et al. reported that SGLT2 inhibitors induced transient hypovolemia and increased RAAS activity within 30 days; however, these values returned to normal levels after 6 months (49). In the current study, dapagliflozin did not increase renin or aldosterone levels at either 1 week or 6 months, suggesting that dapagliflozin might avoid hypovolemia during all treatment periods, mainly due to the increase in compensatory vasopressin secretion, and therefore might contribute to the lack of RAAS activation.

In this study, plasma concentrations of epinephrine and norepinephrine, which are used as markers of sympathetic nervous system activity (56, 57), were significantly lower in the dapagliflozin group than in the control group at 6 months. SGLT2 inhibitors reduce sympathetic nervous system hyperactivity, which may be an underlying mechanism of cardiorenal protection (58–61). However, previous studies have not shown that SGLT2 inhibitors reduced plasma epinephrine and norepinephrine concentrations (62, 63). The possible reason for the difference between the current study and previous studies is the presence or absence of CKD, because the activity of the sympathetic nervous system is increased in CKD patients (64). However, similar to the current study, dapagliflozin significantly decreased the norepinephrine level in kidney tissue and did not change the serum renin concentration in neurogenic hypertensive mice (58). This sympatholytic effect of dapagliflozin may be the reason for the lack of renin and aldosterone increases in this study. Thus, to our knowledge, this is the first report showing that the SGLT2 inhibitor reduced plasma epinephrine and norepinephrine concentrations. From these results, SGLT2 inhibitors may ameliorate sympathetic hyperactivity in patients with CKD.

Loop diuretics have a high risk of hypovolemia, which is linked to the activation of the RAAS, sympathetic nervous system, and adverse cardiorenal outcomes (20, 22, 65). However, interestingly, the addition of SGLT2 inhibitors to loop diuretics reduces all-cause death, readmissions for heart failure, and a composite of cardiovascular death or readmissions for heart failure (65). Furthermore, the cardioprotective effect of SGLT2 inhibitors in patients with heart failure is similar regardless of diuretic use or dose (66, 67). The present study showed that the combined use of dapagliflozin and conventional diuretics prevented hypovolemia in patients without fluid retention and improved hypervolemia in patients with fluid retention. Therefore, the magnitude of changes in body fluid volume induced by SGLT2 inhibitors depends mainly on the baseline body fluid status and not merely on the combination of diuretics. In addition, the combined use of SGLT2 inhibitors and conventional diuretics may prevent excessive counter-regulatory activation of sodium and water reabsorption in the distal nephron (5, 68, 69), potentially strengthening the fluid homeostatic action of SGLT2 inhibitors.

The sustained fluid homeostasis exerted by SGLT2 inhibitors may be a novel mechanism for cardiorenal protection. As both hypervolemia and hypovolemia are risk factors for renal dysfunction and cardiovascular events (27–30), maintenance of euvolemia is a critical treatment strategy for cardiorenal protection. In particular, the sustained inhibition of activated RAAS and the sympathetic nervous system by preventing hypovolemia may contribute to long-term favorable cardiorenal outcomes (16, 53, 70). Therefore, sustained fluid homeostasis by SGLT2 inhibitors shown in the present study is a promising cardiorenal protective effect, likely due to the prevention of activated RAAS and the sympathetic nervous system.

Several limitations of the present study warrant mention. First, this was not a randomized control study and therefore included potential bias or imprecision. Second, the dapagliflozin group includes approximately 3 times as many patients as the control group. This may make the statistical comparisons significantly skewed. Third, the number of enrolled patients was small. However, recent clinical trials focusing on fluid changes induced by SGLT2 inhibitors have also been relatively small-scale (14–45 enrolled patients treated with SGLT2 inhibitors) (26, 36, 45, 47, 48). Fourth, the long-term (more than 6 months) effects of SGLT2 inhibitors on fluid status remain unclear. Fifth, the ethnicity of the patient population was exclusively Japanese. Therefore, the findings may not apply to other ethnic/racial groups.

## 5 Conclusion

The SGLT2 inhibitor dapagliflozin ameliorated fluid retention and maintained euvolemic fluid status in patients with CKD. The compensatory increase in vasopressin secretion may play a critical role in preventing hypovolemia. Additionally, the combined use of SGLT2 inhibitors and conventional diuretics may enhance the fluid homeostatic action of SGLT2 inhibitors. These data suggest that SGLT2 inhibitors exert sustained fluid homeostatic action in patients with various fluid backgrounds.

## Data availability statement

The raw data supporting the conclusions of this article will be made available by the authors, without undue reservation.

## Ethics statement

The studies involving humans were approved by Shin-Oyama City Hospital and Nasu Minami Hosipital. The studies were conducted in accordance with the local legislation and institutional requirements. The participants provided their written informed consent to participate in this study.

## Author contributions

KOk: Conceptualization, Data curation, Formal analysis, Investigation, Writing – original draft. TM: Conceptualization, Data curation, Formal analysis, Funding acquisition, Investigation, Project administration, Writing – original draft. KOh: Data curation, Funding acquisition, Writing – review & editing. MMi: Conceptualization, Data curation, Writing – review & editing. MMo: Data curation, Writing – review & editing. KM: Data curation, Writing – review & editing. YM: Validation, Writing – review & editing. TA: Validation, Writing – review & editing. KS: Validation, Writing – review & editing. DN: Funding acquisition, Validation, Writing – review & editing.
