# Supplementary figures and images for: Fluid homeostatic action of dapagliflozin in patients with chronic kidney disease: the DAPA-BODY Trial
Source: Front Med (Lausanne). 2023 Dec 14;10:1287066. doi: 10.3389/fmed.2023.1287066 (PMC10753517; doi:10.3389/fmed.2023.1287066)

Supplementary Material


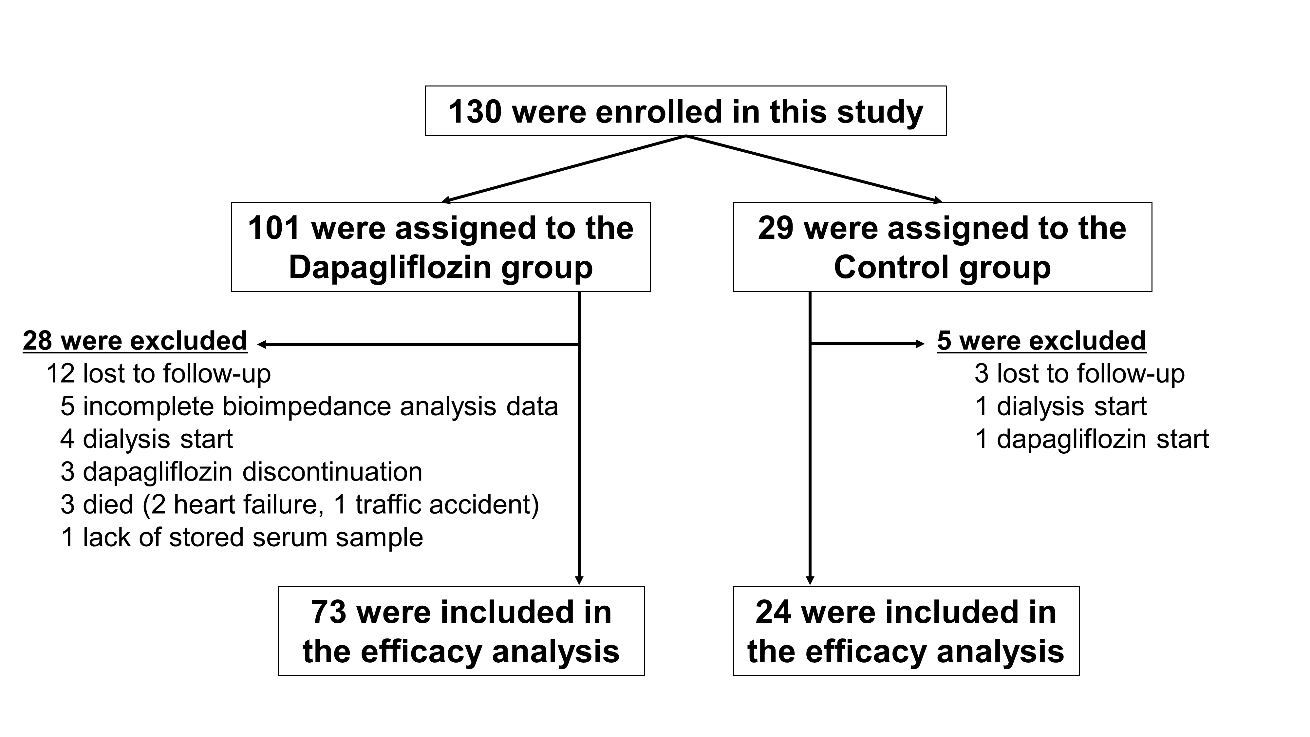


**Supplementary Figure 1.** Patient flow diagram

Supplement: Supplementary file 1 [file Data_Sheet_1.docx]
